# Decoding Analysis of Alpha Oscillation Networks on Maintaining Driver Alertness

**DOI:** 10.3390/e22070787

**Published:** 2020-07-18

**Authors:** Chi Zhang, Jinfei Ma, Jian Zhao, Pengbo Liu, Fengyu Cong, Tianjiao Liu, Ying Li, Lina Sun, Ruosong Chang

**Affiliations:** 1 School of Biomedical Engineering, Faculty of Electronic Information and Electrical Engineering, Dalian University of Technology, Dalian 116024, China; cong@dlut.edu.cn (F.C.); ying.li00@foxmail.com (Y.L.); lnsun@dlut.edu.cn (L.S.); 2 School of Psychology, Liaoning Normal University, Dalian 116029, China; changruosong@163.com; 3 School of Automative Engineering, Faculty of Vehicle Engineering and Mechanics, Dalian University of Technology, Dalian 116024, China; jzhao@dlut.edu.cn (J.Z.); pengboliu@dlut.edu.cn (P.L.); 4 School of Psychology, Shandong Normal University, Jinan 250358, China; 18641077860@163.com

**Keywords:** driver fatigue, alerting effect, EEG, connectivity, clustering, differential entropy

## Abstract

The countermeasure of driver fatigue is valuable for reducing the risk of accidents caused by vigilance failure during prolonged driving. Listening to the radio (RADIO) has been proven to be a relatively effective “in-car” countermeasure. However, the connectivity analysis, which can be used to investigate its alerting effect, is subject to the issue of signal mixing. In this study, we propose a novel framework based on clustering and entropy to improve the performance of the connectivity analysis to reveal the effect of RADIO to maintain driver alertness. Regardless of reducing signal mixing, we introduce clustering algorithm to classify the functional connections with their nodes into different categories to mine the effective information of the alerting effect. Differential entropy (DE) is employed to measure the information content in different brain regions after clustering. Compared with the Louvain-based community detection method, the proposed method shows more superior ability to present RADIO effectin confused functional connection matrices. Our experimental results reveal that the active connection clusters distinguished by the proposed method gradually move from frontal region to parieto-occipital regionwith the progress of fatigue, consistent with the alpha energy changes in these two brain areas. The active class of the clusters in parieto-occipital region significantly decreases and the most active clusters remain in the frontal region when RADIO is taken. The estimation results of DE confirm the significant change (*p* < 0.05) of information content due to the cluster movements. Hence, preventing the movement of the active clusters from frontal region to parieto-occipital region may correlate with maintaining driver alertness. The revelation of alerting effect is helpful for the targeted upgrade of fatigue countermeasures.

## 1. Introduction

Fatigue is a phenomenon that reduces and weakens physical, mental, and emotional application and often entails decreased physiological functions (including speed, strength, coordination, reaction time, balance, and decision) [1]. In the transportation system, fatigue decreases drivers’ alertness level and gives rise to road accidents. According to related road crash investigations, about 10%–20% of all crashes and 40% of fatal crashes are caused by driver fatigue [2,3,4,5]. Car crash is the number one reason for teenage death in the U.S., and driver fatigue is reported to be responsible for more than 57% of traffic accident death [6].

Against the background above, safe and affordable countermeasures are worthy of being explored. In real-world driving conditions, drivers take various countermeasures to relieve fatigue and maintain mental and physical performance, such as caffeine consumption, napping, opening a window, listening to the radio, talking, and parking to take a walk. Among them, opening a window and listening to the radio are considered to be effective ways to stay awake by most drivers (professional and nonprofessional) [7]. A previous study restricted young adult drivers’ sleep to 5 h at night and exposed them to cold air (AIR), listening to the radio (RADIO), or nil treatment during driving [8]. When comparing the different countermeasures, RADIO made the drowsiness level represented by Karolinska Sleepiness Scale (KSS) score significantly decrease. Nevertheless, the effect of AIR is transient and non-significant. Even though RADIO has been proven to be a relatively effective “in-car” countermeasure, the brain mechanism underpinning its alerting effect is still unknown. This paper aims to develop a method to explore the RADIO effect so that the precision of countermeasures can be improved to maintain driver alertness more effectively.

An important advancement in neuroscience research, reflecting the modern concept of the brain as a highly integrated and dynamic system, is the assessment of functional connectivity in brain networks (i.e., increase or decrease of correlation from brain activities in different areas) [9]. Changes in functional connectivity across mental states can provide richer information about human cognition than simpler univariate approaches [10], while communication between brain circuits is also reflected in neural oscillations, which can be measured through spectrum analysis of electroencephalography (EEG) recordings in frequency domain [11]. In the frequency domain, previous studies have demonstrated that the power of alpha and theta oscillations predominantly in the central and posterior brain regions (parietal-occipital) is generally increased when the subjects are fatigued or tired, in contrast to a decrease in higher frequency bands [12,13]. Therefore, the oscillatory information, especially alpha-band indicator, has been considered as one of the most prominent EEG-indicators [14]. Alpha-band power (8–13 Hz) has been shown to be sensitive to the early stage of fatigue and was applied in fatigue assessment by various real-traffic and simulated driving studies. Recent research has demonstrated that alpha spindles, which consist of short (0.5–2 s) bursts of high frequency alpha activity, in the parietal/occipital area are statistically related to fatigue and drowsiness [15]. In the aspect of connectivity analysis, several studies have reported that the functional brain networks become more integrated during task performance in comparison with the resting state, but linearly decreased with ongoing time-on-task [10,16]. In the prolonged visuomotor vigilance task, Gaggioni et al. [17] suggested that decreased propagation of EEG response evoked by transcranial magnetic stimulation within the fronto-parietal cortex was related to the failure of increased vigilance. Under the simulated driving condition, Kong et al. [18] also revealed the degraded performance of small-world features of brain networks under a fatigue state, providing further support for the presence of a reshaped global topology in connectivity networks when drivers shifted from the alert to drowsy stage. Zhao et al. [19] attributed the shift to a more economic but less efficient configuration, or an inability to retrieve these resources related to mental fatigue. Obviously, the interaction analysis of functional connectivity and the oscillations like alpha wavescan provide more adequate and reliable information for exploring the neural mechanism of the alerting effect of RADIO.

However, the connectivity analysis of time series will encounter significant challenges in practical applications. Traditional approaches of connectivity analysis, which can be used to estimate the changes of signal coupling across mental states, usually characterize either different single connections or the system’s average behavior [20]. Recent studies have shown that these approaches ignore complex spatiotemporal patterns of activity, for example global signal changes to propagating waves [21,22,23]. Additionally, the connectivity analysis from sensor space data isnon-robust and it is sometimes difficult to obtain results that are statistically significant, to reject the null hypothesis in cognitive studies. This issue is also serious for the measure of EEG, which records brain electrical activities non-invasively at a millisecond time scale. Owing to signal mixing, which translates to volume conduction in the case of EEG recordings and to signal leakage in source reconstructed EEG data, the activity of any single neuronal source is detected by a spatially widespread group of sensors [24]. From the perspective of individual sensors, the same sensor may also pick up multiple sources. Thus, EEG-based connectivity analysis suffers from two types of false positives—artificial and spurious interactions. Artificial interactions directly caused by signal mixing neglecting real interactions between neuronal groups at the considered locations can be reduced by a number of binarized connectivity matrices that typically aim to remove linear coupling terms [25]. Spurious interactions (referred to as ghost interactions) arising from the leakage of signals from a true link of sources to the surrounding links are more difficult to be processed, because of multivariate mixing effects [26,27]. Up to now, steps towards addressing the problem have been taken for suppressing spurious interactions, such as oscillation-based and phase-based estimates [28,29]. Even though some improvements are achieved, all the interaction measures are still sensitive to spurious interactions with residual signal mixing in source space [24].

In this context, we are not seeking to further suppresss purious interactions, but to mine effective information with the false positives. A single link in a brain network may be spurious link, but we view the problem from a different angle. Two distinct groups of connections with spurious links can be classified by machine learning method automatically. As signal mixing do not vary over time [25,29,30,31], the change of pattern classification of connection groups over time is considered to reflect the shift of mental states. In this study, the smallest group of connections is defined as a connection cluster composed of a node and all the connections linked to it. Since the definition includes node, the classification changes manifest the evolution of spatiotemporal patterns. Accordingly, we propose the clustering algorithm implemented in brain networks. In order to verify that the spatiotemporal evolution indeed generates the information content change, entropy is calculated in different brain areas after clustering. In information theory, entropy represents complexity and uncertainty of information source and embodies information content through the probability distributions that underlie the process of communication [32]. As a nonlinear estimation of dynamical EEG activity, entropy-based algorithms have been proven to be useful and robust estimators for evaluating regularity or predictability [26,33,34]. For example, Shi et al. [35] utilized differential entropy (DE) to extract the EEG features of driver alertness and found it was more accurate and stable comparing with energy spectrum, autoregressive parameters, fractal dimension, and sample entropy. Therefore, DE was selected to implement the entropy estimation. 

This paper is organized as follows. In Section 2, we describe the experimental details and EEG data preprocessing to extract alpha oscillations (see Section 2.1 and Section 2.2), illustrate the functional connectivity analysis to construct brain networks (see Section 2.3), explain clustering algorithm implemented in the constructed brain network (see Section 2.4), elaborate the evaluation method of information content based on DE (see Section 2.5), and provide the statistical analysis process (see Section 2.6). Results of the study are presented in Section 3 and discussed in Section 4.

## 2. Materials and Methods

**Figure 1:**
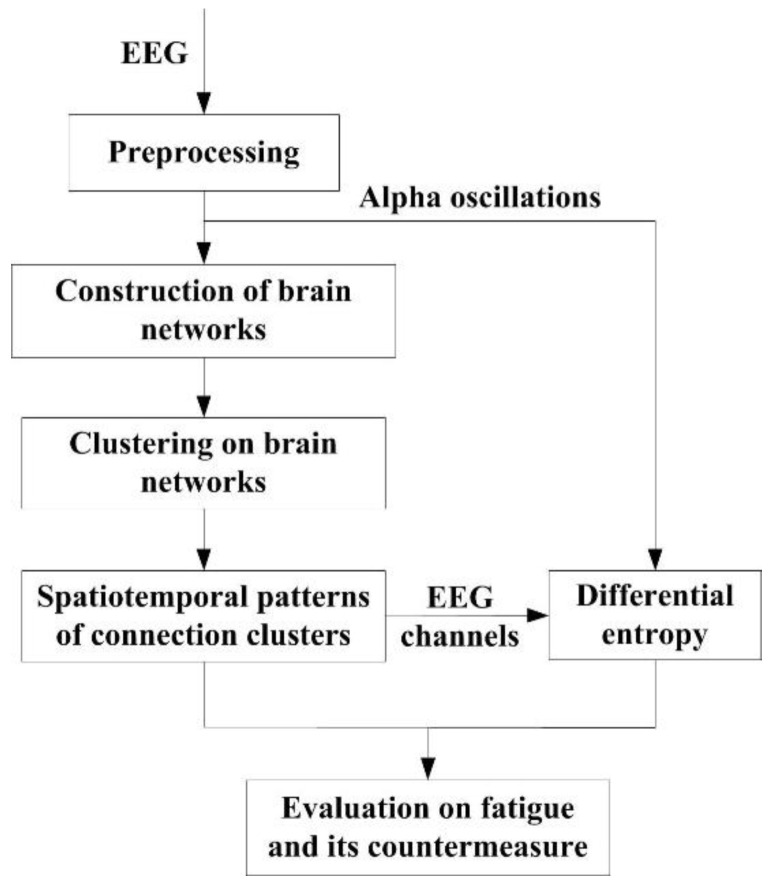
Flowchart of the data-driven methodology.

The signal flowchart of the proposed methodology is shown in Figure 1. The analysis is based on clustering algorithm implemented in brain networks and entropy assessment in alpha band. The EEG data come from the prolonged driving experiments described in Section 2.1. In frequency domain, alpha oscillations are extracted by discrete wavelet transform, which is included in data preprocessing (see Section 2.2). In spatial dimension, the brain networks of alpha oscillations are constructed based on connectivity estimation in alpha band (see Section 2.3). The nodes of the classified clusters are extracted through the hierarchical clustering algorithm so that labels of classification outputs manifest the enrichment levels of alpha interactions (see Section 2.4). Clustering evolved at the data of different stages brings spatiotemporal changes. Finally, according to the spatiotemporal patterns, the statistical analysis of DE in alpha band is made in corresponding brain areas (see Section 2.5). The spatiotemporal patterns and DE values are used to assess the alerting effect of the fatigue countermeasure. 

### 2.1. Experiments and Data

**Figure 2:**
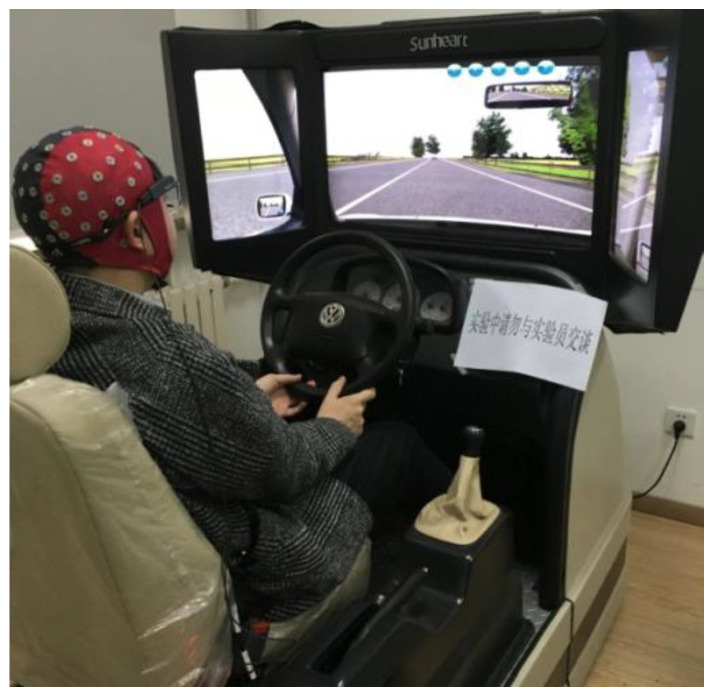
Experimental setting.

This study was conducted in accordance with the Declaration of Helsinki, and the protocol was approved by the Ethics Committee of Dalian University of Technology (protocol number: 2018-040). All subjects gave their informed consent for inclusion before they participated in the experiments. Twenty-eight healthy subjects aged from 20 to 30 years old were recruited in the study. None of the subjects reported mental disorders or neurological disorders. They had normal sleeping patterns, normal vision or corrected to normal with glasses or contact lenses, without prescription, without alcohol and caffeine 24 h before the experiment. Each subject drove along at daytime for more than one hour under monotonous highway environment produced in a driving simulator (QJ-3A1). The interactive display system of the simulator was developed based on virtual reality (VR). The VR scenes were displayed on the car-like three-sided screen with an angle of 120° (see Figure 2). The subjects were divided into the following two groups according to whether the countermeasure was implemented.

The control group was composed of 14 male subjects with a mean age of 25.64 years. In the control group, no countermeasure was taken. The subjects just drove under the simulated driving environment to induce fatigue.

The experimental group was also composed of 14 male subjects with a mean age of 22.57 years. In the experimental group, the subjects were exposed to RADIO during driving. They chose the talk show radio station that they would normally select on such journeys. Volume was at their discretion.

Nineteen standard electrodes (i.e., Fp1, Fp2, F7, F3, Fz, F4, F8, T3, C3,Cz, C4, T4, T5, P3, Pz, P4, T6, O1, and O2) mounted on a cap (eego™mylab from ANT Neuro, Berlin, Germany) were attached to the scalps following the International 10–20 System to collect the EEG data of the subjects during driving. The EEG’s sampling frequency is 500 Hz.

### 2.2. Data Preprocessing

The original EEG signals are inevitably disturbed by a large quantity of high frequency and low frequency noise (i.e., artifact), such as power frequency noise, body movement and blinking, which needs to be removed. In addition, various aspects of the signals, such as trends, sudden changes, and start/end tag information, may be useful for alerting effect analysis in the time domain and need be retained. Wavelet transform is an effective tool to analyze the various components of a non-stationary signal due to its attractive properties such as good local representation in both time and frequency domain and multi-rate filtering (differentiating the signals having various frequencies) [36]. Referring to [37], the raw EEG data were filtered by wavelet decomposition with 9 levels; after that, wavelet coefficients (7.8–15.6 Hz) at fifth level were used to reconstruct alpha waves. Moreover, the wavelet-based threshold technique in [26] was used to correct the filtered signals.

On account of the staged and accumulative properties of fatigue [38,39], the continuous data after artifact removal were epoched into segments of 10min. Segments 1–6 correspond to the data within 0–10 min, 10–20 min, 20–30 min, 30–40 min, 40–50 min, and 50–60 min, respectively.

In the frequency domain, alpha ratio was calculated in each segment and group to realize the oscillation analysis. The alpha ratio is defined as the energy of alpha frequency band divided by the total energy of EEG frequency band. Therefore, it reflects the change of alpha activity level.

### 2.3. Construction of Brain Networks

In accordance to graph theory [40], a brain network can be represented by a graph G(N,E), where N and E are the node and edge (or link) sets, respectively. We assigned EEG electrodes to the elements of N. The edges reflecting the adjacency relations among the nodes in the networks can be described by the functional connectivity matrix *F,* whose element *F*(*i*,*j*) shows the edge between nodes (electrodes) *i* and *j*. To show that the effective information of the networks on maintaining driver alertness can be dug out with spurious interactions, the cross-correlation method, which has been proven to suffer from primary and secondary leakage [24], is adopted in the estimation of edges. The correlation between the EEG signals *s_i_* and *s_j_* can be calculated by the following equations:

(1) $$\gamma_{ij}=\left|\frac{CC(s_{i},s_{j})(\tau )}{\sqrt{CC(s_{i},s_{i})(0)CC(s_{j},s_{j})(0)}}\right|,$$

(2) $$CC(s_{i},s_{j})(\tau )=\{\begin{array}{c} \sum_{t=1}^{N-\tau }s_{i}(t+\tau )s_{j}(t),\tau \geq 0\\ CC(s_{j},s_{i})(-\tau ),\;\;\;\tau <0 \end{array},$$

where time delay *τ* is set to 0. *γ_ij_* corresponds to the element of the functional connectivity matrix *F*, which presents in *i*th row and *j*th column. To exclude self-connections of nodes, the elements on the main diagonal of *F* are set to zero. The other elements of *F* reflect functional coupling (i.e., functional connectivity) of the signals in different channels. Here, the signals were selected as the alpha oscillations after preprocessing to construct alpha oscillation networks.

As a measure of overall connectivity strengths in a brain network, global connectivity energy has been used to assess fatigue during driving. The previous studies have demonstrated that its staged change is associated with the accumulation of fatigue [41,42]. The global connectivity energy is defined as the total energy of all the connectivity matrix elements. In this work, the global connectivity energy was calculated to compare the alertness level both between the segments and between the two groups in the experiments.

### 2.4. Clustering on Brain Networks

In the constructed brain networks, an unsupervised clustering algorithm was employed to search the significant connectivity information at group level (connection cluster). Since every connection cluster only has a node, the classification of the clusters equals the classification of their nodes in a brain network. Thereafter, the connections of a connection cluster are defined as the attributes of its node.

Mathematically, we re-express the constructed brain network *G*(*N*,*E*) with *N*nodes as *X* = {*X*_1_, *X*_2_, …, *X_N_*}^T^, where *X_i_* ={$x_{i}^{1}$, $x_{i}^{2}$, …, $x_{i}^{M}$} with *M* links (*M* = *N* − 1 for weighted network) is a vector denoting the *i*th node whose attribute $x_{i}^{j}=F(i,j)$ (*j* ≠ *i*) is a scalar representing the strength of the functional connectivity between Node *i* and Node *j*. The spatial connectivity information is searched based on agglomerative hierarchical clustering, which establishes the foundations for inducing a hierarchical clustering from a newly represented, or newly encoded, mapping of functional connectivity matrix *F*.

To begin with, each node and the connections linked to it in a constructed brain network are considered as a single-element cluster group at the lowest level, i.e., *C_i_* = {*X_i_*}, 1 ≤ *i* ≤ *N*. Dendrogram*_k_* = {*C*_1_, *C*_2_, …, *C_N_*}, *k* = *N*. Then, based on the distance (similarity function) calculation, two closest cluster groups are successively merged to reduce the number of cluster groups by 1 until a single cluster group remains at the highest level (i.e., *k* = 1):

(3) $$(a,b)=argmin_{\left(i,j\right)}\{d(C_{i},C_{j}):\;1\leq i<j\leq k\},$$

(4) $$\mathrm{merge}(C_{a},C_{b})=C_{2N+1-k},$$

(5) $$Dendrogram_{k-1}=Dendrogram_{k}-\{C_{a},C_{b}\}+\{C_{2N+1-k}\},$$

where *C_i_* and *C_j_* denote two different clusters; *k* is the level number; Dendrogram indicates the cluster set and its element number is reduced by 1 to reach a higher level in Equation (5); and *d* represents Euclidean distance between two clusters. This Euclidean distance can be calculated by the following expressions:

(6) $$d(C_{i},C_{j})=d(X_{a},X_{b}),$$

(7) $$d(X_{a},X_{b})=\sqrt{\sum_{m=1}^{M}{\left(x_{a}^{m}-x_{b}^{m}\right)}^{2}},$$

where *M* denotes the number of attributes of the node vectors ***X****_a_* and ***X****_b_*. $x_{a}^{m}$ and $x_{b}^{m}$ indicate the attributes (corresponding to functional connectivity) of the two node vectors.

Finally, we cut the dendrogram to complete clustering on brain networks to obtain different node groups (i.e., cluster groups) of which number (*Z*) is predetermined. As the number of the cluster groups contained in Dendrogram is equal to the level number *k*, *Z* cluster groups will be left by the *Z*th level cut through the dendrogram. That means we classify the nodes of the brain networks into *Z* groups according to the elements of Dendrogram*_Z_*.

**Figure 3:**
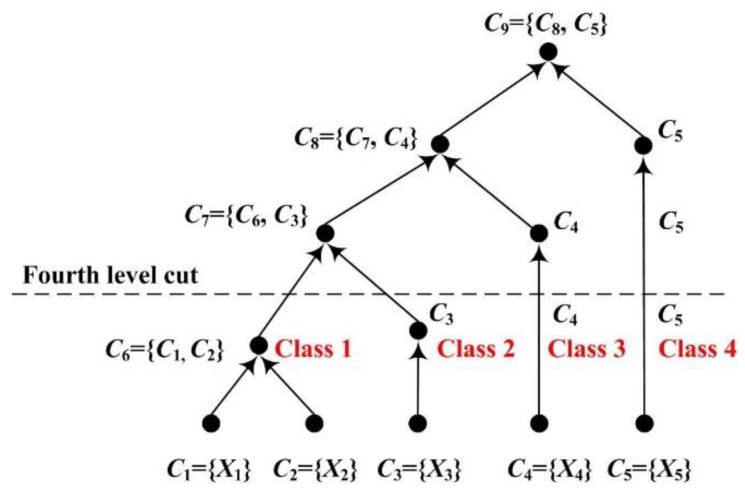
An exemplar dendrogram representing a possible hierarchical clustering process for five connection clusters.

In this study, *Z* is set to 4 to acquire class pattern changes associated with fatigue in the control group and alerted the effect of RADIO in the experimental group. As shown in Figure 3, four cluster groups are left by the fourth level cut. Because the cluster groups are merged based on the distance, the later the cluster group is involved in the merged operation, the more different the cluster group will be. The four cluster groups (i.e., *C*_6_, *C*_3_, *C*_4_ and *C*_5_) are labeled as Class 1, Class 2, Class 3, and Class 4 with the increase of difference.

### 2.5. Differential Entropy

DE is considered as a univariate and non-linear measure of information content in a given time series *X*. It quantifies information in an arbitrary time series *X* of length *N* as the probability that the value *x* of *X* within a certain area. The probability is represented by the integral of the probability density function in the area. Its calculation formula can be expressed as:

(8) $$DE(X)=-\int_{X}p(x)\log p(x)dx,$$

where *X* represents the time series, *p*(*x*) denotes the probability density function of *X*.

In this work, the alpha oscillations after preprocessing were used as the time series to calculate the *DE* values. *DE* was performed on all the nodes of the brain networks constructed in Section 2.3. The nodes which were used for *DE* statistical analysis was determined by the spatiotemporal patterns of connection clusters.

### 2.6. Statistical Analysis

To compare alertness evaluation performance, the above EEG features (i.e., global connectivity energy, alpha ratio, and *DE*) of the subjects in the control and experimental groups were averaged for each driving stage (Segments 1–6). The standard deviations were calculated to measure their variability (see Figures 6 and 7 and Table 3). When the staged changes of feature means are acquired, it needs to be confirmed whether the observed difference between group means reflects actual difference in the sample population of both groups. The Wilcoxon rank-sum test, also called the Mann–Whitney U test, is a non-parametric test used to determine whether two independent sample populations follow the same distribution [43]. It does not require the data normality assumption and is suitable for small samples (such as subject number <30). Hence, the Wilcoxon rank-sum test was selected for statistical analysis to check whether the EEG features can reflect the actual differences between the two groups in Section 2.1. The significance level *α* was set to 0.05. Values of *p* < 0.05 (with a confidence interval of 95%) indicate that the samples in the two groups come from different distributions—the group means are different.

## 3. Results

**Figure 4:**
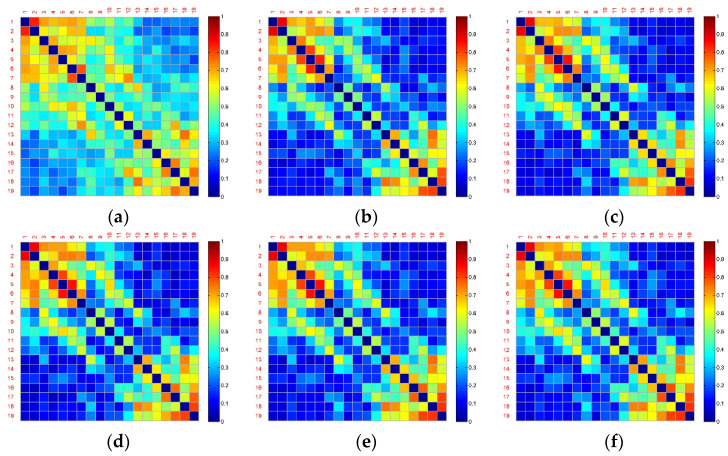
Averaged functional connectivity matrices of the control group. (**a**) Functional connectivity matrix in Segment 1; (**b**) Functional connectivity matrix in Segment 2; (**c**) Functional connectivity matrix in Segment 3; (**d**) Functional connectivity matrix in Segment 4; (**e**) Functional connectivity matrix in Segment 5; (**f**) Functional connectivity matrix in Segment 6.

**Figure 5:**
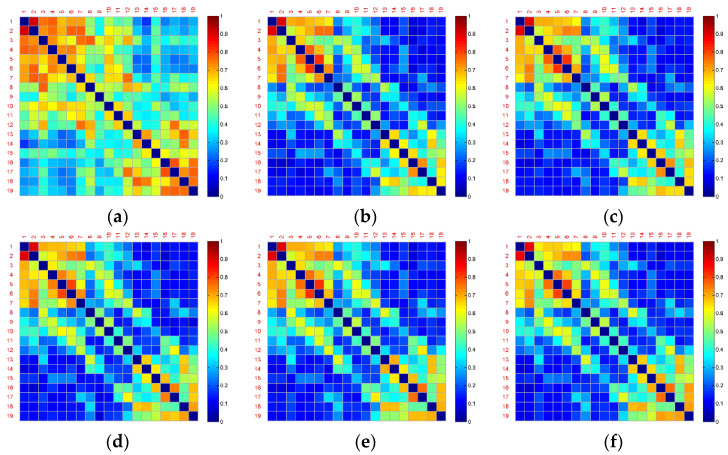
Averaged functional connectivity matrices of the experimental group. (**a**) Functional connectivity matrix in Segment 1; (**b**) Functional connectivity matrix in Segment 2; (**c**) Functional connectivity matrix in Segment 3; (**d**) Functional connectivity matrix in Segment 4; (**e**) Functional connectivity matrix in Segment 5; (**f**) Functional connectivity matrix in Segment 6.

Figure 4 and Figure 5 show the averaged functional connectivity matrices of the control and experimental group across the subjects, respectively. Nodes Fp1, Fp2, F7, F3, Fz, F4, F8, T3, C3, Cz, C4, T4, T5, P3, Pz, P4, T6, O1, and O2 are numbered 1–19. Each small square surface in a matrix represents a connection between two nodes. Color indicates the value of the correlation coefficient reflecting connectivity strength. The correlation coefficients on the main diagonal were set to zero (deep blue) to exclude self-connection. All the functional connectivity analyses are based on the functional connectivity matrices. Traditional approaches of connectivity analysis suffer from spurious interactions. As demonstrated in Figure 4 and Figure 5, from Segment 1 to Segment 6, the changes in vigilance affect the functional connectivity. The overall connectivity strength of both the control and experimental groups becomes weaker (more blue surfaces) after Segment 1. However, there is almost no change after Segment 1 and no difference between inter- and intra-groups through observation.

**Figure 6:**
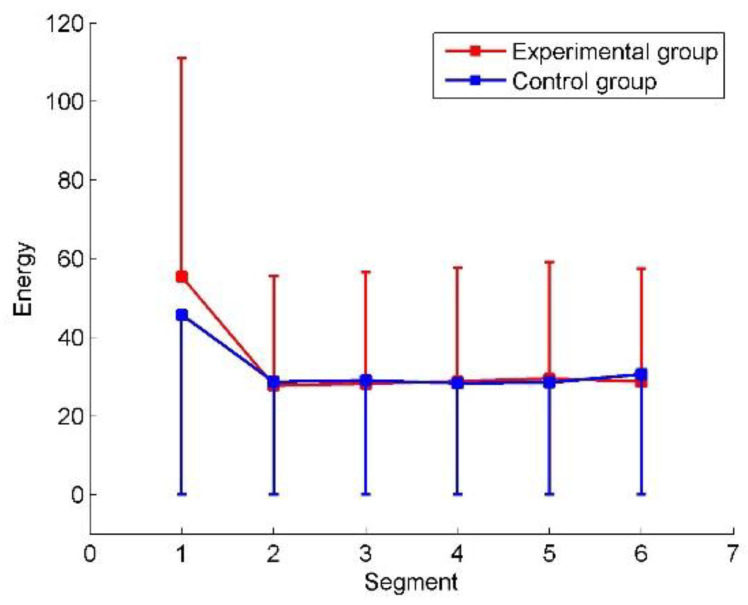
Global connectivity energy. The averages and standard deviations are plotted.

The global connectivity energy was used to measure the overall functional connectivity of each brain network in different stages. The energy of all the elements of the functional connectivity matrix *F* was calculated and the results of statistical analysis are shown in Figure 6. Both the experimental group and control group curves of the averaged global connectivity energy decline in the second segment. Table 1 presents the results of the Mann–Whitney U test for global connectivity energy. There is no statistically significant difference between the two groups, but with large standard deviations for the subjects in each group. It illustrates that the networks’ average behavior is largely affected by individual differences of the subjects. The pivotal information of fatigue and alerted effect of the countermeasure is concealed.

One of the reasons for these poor results is that the brain networks may contain false positives. For example, it is uncertain which connections are true interactions among the red surfaces in the top left corner in Figure 4b. Nevertheless, the blue surfaces and red surfaces in the top left and in the bottom right corner of this figure can be classified into different groups by observation. Therefore, dividing into subsets of electrodes may obtain a more powerful result.

**Figure 7:**
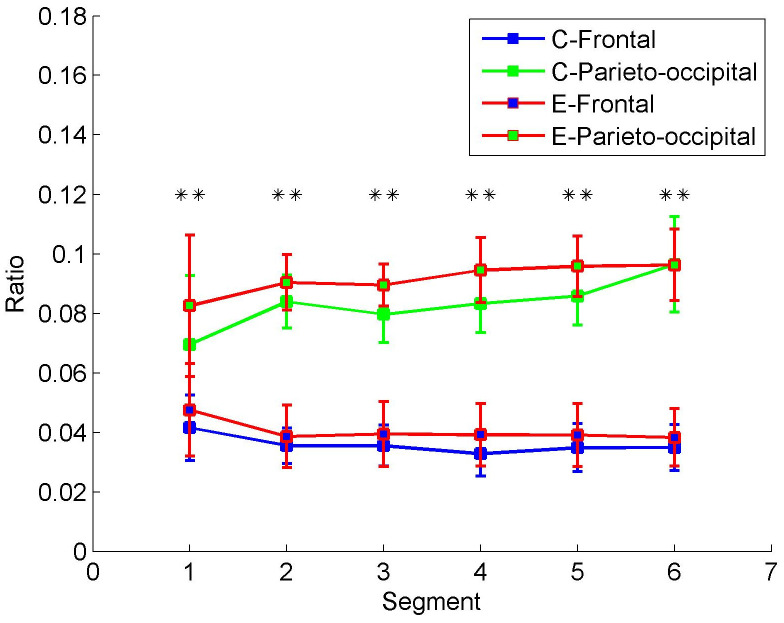
Alpha energy ratios in the control and experimental groups. The averages and standard deviations from the subjects are plotted. C-Frontal and C-Parieto-occipital represent the frontal and parieto-occipital ratios in the control group. In contrast, E-Frontal and E-Parieto-occipital represent the frontal and parieto-occipital ratios in the experimental group. ** denotes *p* < 0.01.

Figure 7 confirms that the activities of different brain regions (nodes) can be classified. After wavelet decomposition implemented in data preprocessing, the wavelet coefficients in the alpha band and the total band of EEG can be obtained (refer to Section 2.2). To quantify alpha activity level in EEG, the energy of the wavelet coefficients in alpha band was calculated and converted to energy ratio, which was divided by the total energy of EEG band. Figure 7 shows the energy ratios of two brain areas in the control and experimental groups. The frontal and parieto-occipital ratios are acquired by averaging ratios across the frontal channels (i.e., Fp1, Fp2, F7, F3, Fz, F4, and F8) and parieto-occipital channels (i.e., T5, P3, Pz, P4, T6, O1, and O2), respectively. In contrast with the increase of parieto-occipital ratios, the frontal ratios drop gradually in both the control and experimental groups from Segment 1 to Segment 6. As shown in Table 2, the Mann–Whitney U test revealed that the alpha energy ratios between frontal and parieto-occipital regions were significantly different in every segment. The alpha energy ratios with significant differences between the two regions were marked with asterisks in Figure 7. The mark ** represents *p* < 0.01. Therefore, the frontal and parieto-occipital ratios can be classified into two categories over time in both the control and experimental groups. However, the differences between the control and experimental groups are not significant (see control group vs. experimental group in Table 2). Hence, simply dividing into the subsets of electrodes is not enough. The differences between the control and experimental groups are explored by the proposed clustering algorithm and DE.

**Figure 8:**
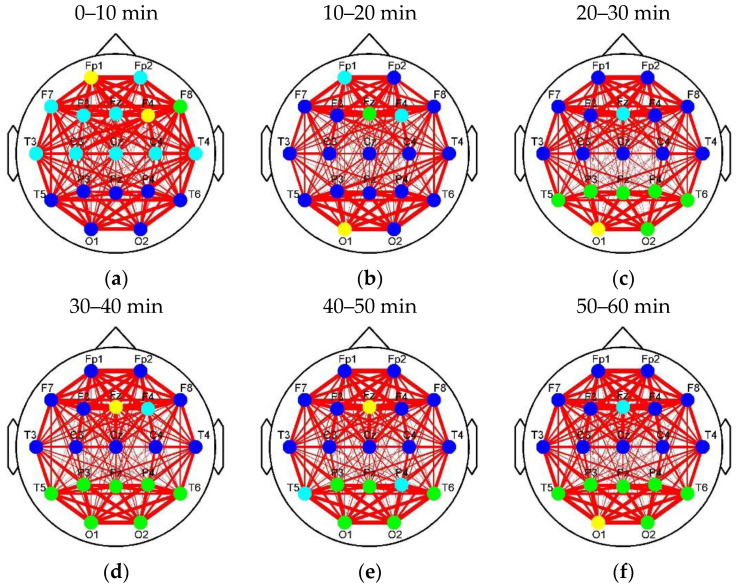
Classification of connection clusters in each stage brain network of the control group. (**a**) Classification in Segment 1; (**b**) Classification in Segment 2; (**c**) Classification in Segment 3; (**d**) Classification in Segment 4; (**e**) Classification in Segment 5; (**f**) Classification in Segment 6. Classes 1–4 was represented by blue, cyan, green, and yellow, respectively.

**Figure 9:**
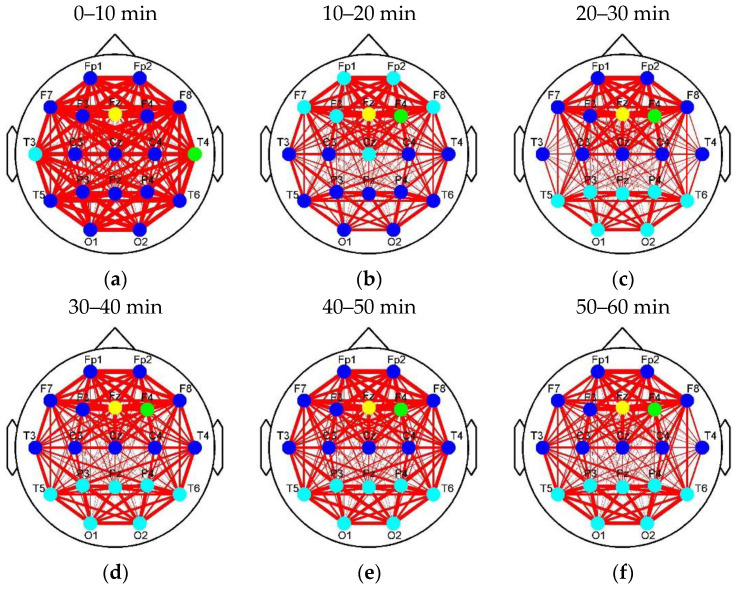
Classification of connection clusters in each stage brain network of the experimental group. (**a**) Classification in Segment 1; (**b**) Classification in Segment 2; (**c**) Classification in Segment 3; (**d**) Classification in Segment 4; (**e**) Classification in Segment 5; (**f**) Classification in Segment 6. Classes 1–4 was represented by blue, cyan, green, and yellow, respectively.

Figure 8 and Figure 9 show the clustering results of the brain networks in the control and experimental groups, respectively. The element size of functional connectivity matrices in Figure 4 and Figure 5 is represented by the link thickness of the brain networks. Every node represents a connection cluster. Different colors (blue, cyan, green, and yellow) on nodes indicate different classes (Classes 1–4). As the hierarchical clustering algorithm was adopted, the connection clusters with the shortest distance between each other (blue nodes) were classified as Class 1 first, and the yellow nodes whose attributes were ‘farthest’ from others were finally sifted out and labeled as Class 4 by using Equations (3)–(7). That means that the higher the class is, the more different or active the cluster will be. Since the subjects in the control group drove without any extra stimulus from countermeasure and induced fatigue in Segment 1, the brain network with the classification of the connection clusters presented in Figure 8a was considered as the default mode network of driving in the experiments of this study (DMND). Compared to DMND, in other segments of Figure 8, strong connections are concentrated in the frontal and parieto-occipital regions and the connectivity between the frontal and parieto-occipital regions significantly reduces, which corresponds to the trend in Figure 6. Particularly, the classification changes of the connection clusters (or nodes) in the brain networks are staged with fatigue accumulation. As shown in Figure 8a–f, the nodes labeled as high classes (i.e., cyan, green, and yellow nodes) move to the parieto-occipital region step by step. In Figure 9a, the nodes (T3, T4, and Fz) in the auditory and frontal areas of brain are labeled as Class 2–4 (high classes), respectively, when the drivers were exposed to RADIO. It reveals there are intermediately active clusters in the auditory area. Hence, the brain network in Figure 9a was considered as the default mode network of RADIO (DMNR). Compared to DMNR, superficial connectivity in other segments of Figure 9 seems to return to fatigue mode with weak links between the frontal and parieto-occipital regions. In addition, the cyan nodes move to the parieto-occipital region. However, the most active clusters labeled as Classes 3 and 4remain in the frontal area from Segment 2 to 6.

**Figure 10:**
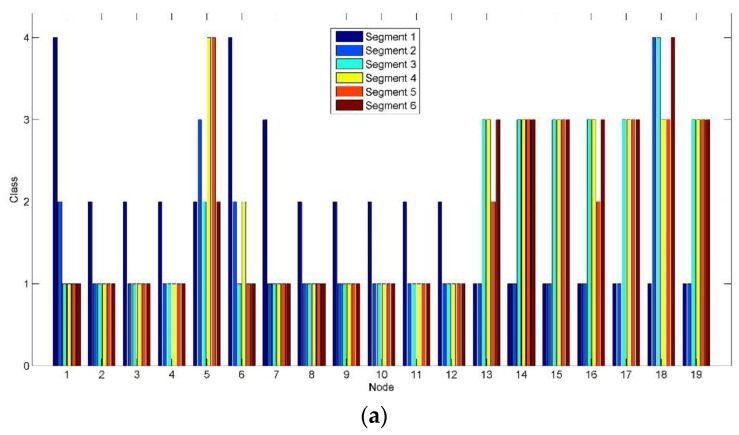

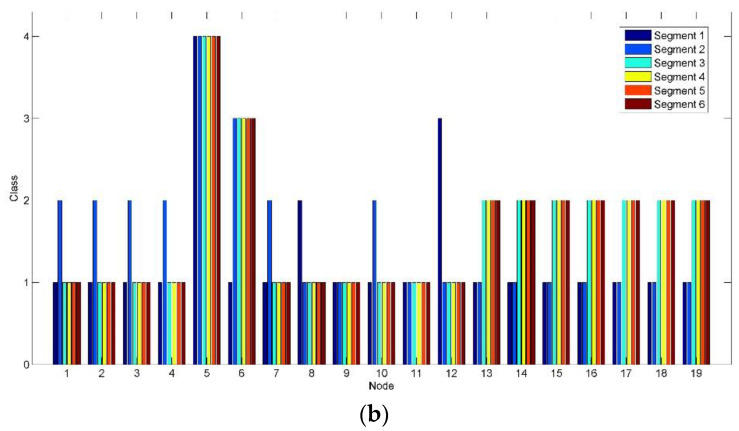
Class sets from all the nodes and segments. (**a**) Class set of the control group; (**b**) Class set of the experimental group. Nodes Fp1, Fp2, F7, F3, Fz, F4, F8, T3, C3, Cz, C4, T4, T5, P3, Pz, P4, T6, O1 and O2 are numbered 1–19.

Figure 10 shows the class change on each node corresponding to Figure 8 and Figure 9. In Figure 10a, high class (Class 3) is mainly marked in the parieto-occipital region (Nodes 13–19) by the clustering algorithm implemented in brain networks after Segment 2. Compared to Figure 10a, the active level of the most clusters (represented by the nodes) of the parieto-occipital region in Figure 10b reduces by one grade to Class 2. By contrast, Nodes 5 and 6 in the frontal region maintain high class. It reveals a shift tendency of the active connection clusters constructed based on alpha oscillations from the parieto-occipital region to the frontal region, when RADIO was taken during the prolonged driving task.

**Figure 11:**
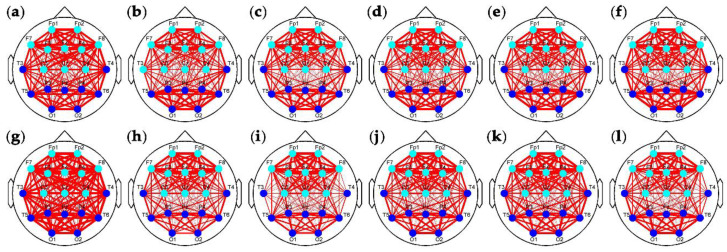
Community detected by Louvainalgorithm. (**a**–**f**) Community detected in Segments 1–6 of the control group; (**g**–**l**) Community detected in Segments 1–6 of the experimental group.

To demonstrate the superiority of the proposed clustering method, the widely used Louvain algorithm was adopted to detect the community in the brain networks. According to the community, the subsets of electrodes can also be distinguished. However, because too much connectivity information is confused (see Figure 6), there is no change in the community division over time in both the two groups in Figure 11, if the community change of T3 in Figure 11b is neglected.

**Figure 12:**
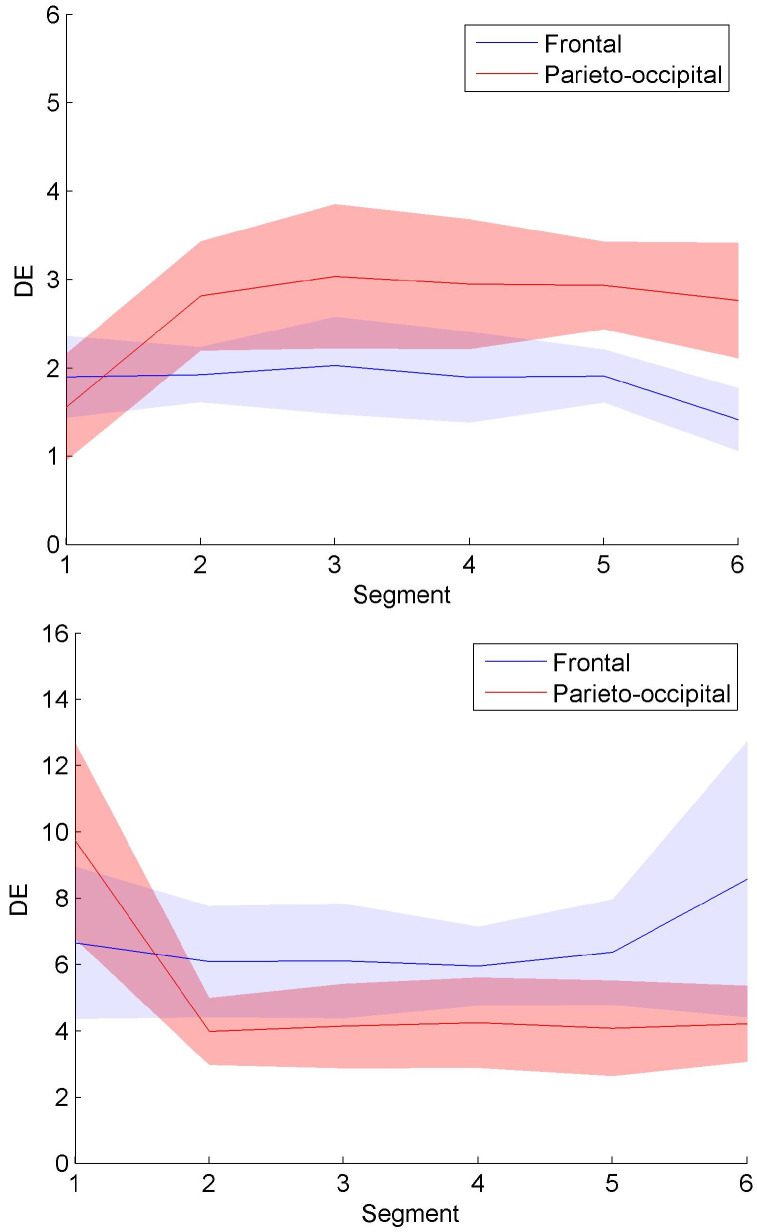
DE change during driving. (**a**) Averaged DE across subjects in the control group; (**b**) Averaged DE across subjects in the experimental group. All the DE values of the subjects in each group were averaged and the shaded area indicates the standard deviation.

Figure 12 confirms that the spatiotemporal evolution of connection clusters in Figure 8 and Figure 9 generate the information content change. DE was calculated on all the nodes in Figure 8 and Figure 9. According to the movements of connection clusters between frontal and parieto-occipital regions, DE values were averaged across Nodes Fp1, Fp2, F7, F3, Fz, F4, and F8 and across Nodes T5, P3, Pz, P4, T6, O1, and O2 to quantify information content of frontal and parieto-occipital regions, respectively. Figure 12 presents the averaged DE curves across subjects in each group. The shaded area indicates the standard deviation. As shown in Figure 12a, the trend of the frontal entropy values in the control group is declining over time, whereas the parieto-occipital entropy curve has a significant upward trend. Overall, the DE values of the experimental group in Figure 12b presents the opposite trend compared with Figure 12a. The entropy values in Figure 12, which were expressed as mean±standard deviation (SD) across the subjects, are presented in Table 3. As shown in Table 3, the average entropy values of the experimental group are larger than those of the control group. Through the Mann–Whitney U test, the DE differences between groups present statistical significance for all the segments (see Table 4). The DE values with significant differences between groups were marked with asterisks in Table 3. The asterisks * and ** indicate *p* < 0.05 and *p* < 0.01, respectively. Since all the *p*-values are less than 0.05, the proposed method contributes to the evaluation of alerting effect in the experimental group.

## 4. Discussion

Fatigue is multifaceted in nature [44]. It not only brings the simple increase of alpha activity level and decoupling between different brain regions, but also changes wave propagation, hemispheric symmetry, and modular network properties. In Figure 7, alpha energy ratios in the parieto-occipital region presented a gradual increase trend during the prolonged driving task, which was consistent with previous studies, where oscillatory activity at alpha band had been observed to increase in the posterior brain regions (parietal-occipital) during attentional lapses [45] and during states of drowsiness relative to states of alertness [14,15]. As is known to us, alpha oscillations occur during wakefulness, particularly over the occipital cortex, appear markedly at eye closure and decrease at eye opening [46]. Thus, the increase of alpha energy ratios probably indicates more eye closures and driver drowsiness. Nevertheless, the alpha energy ratios of frontal region showed the opposite trend. The frontal region is involved in higher cognitive functions, such as working memory, selective attention, error detection, and behavior control [47,48,49]. The opposite trend in the frontal region may correspond to the weakening of motivation and wakefulness. It seems that there was an alpha wave propagation between the frontal and parieto-occipital regions. As one fell, another rose.

However, for the analysis of functional connections, a wide variety of information underlying fatigue and alerting effect of the fatigue countermeasure was still implicit, due to false positives. As shown in Figure 4, Figure 5 and Figure 6, the strong connectivity in Segment 1 may just reflect the difference of the initial state. The curve of the averaged global connectivity energy was relatively flat after Segment 1, which cannot reveal the fatigue information or concur with the fatigue assessment of alpha energy ratios. More importantly, it lost the countermeasure information without statistically significant difference at intergroup. Previous studies have already proven the effectiveness of RADIO as an “in-car” countermeasure [8]. Such information must be involved in the experimental group somehow but implicated by signal mixing.

Under the condition of no significantly statistical difference using the traditional connectivity analysis (see Figure 4, Figure 5 and Figure 6), the proposed methodology was applied to mining the spatiotemporal information (see Figure 8, Figure 9 and Figure 10 and Figure 12). Particularly, we employed clustering algorithm performed in the brain networks with false positives to try to point out what happened in the brain after RADIO was taken. We considered that all the links connected to a node was basic unit (connection cluster) of connectivity analysis, not a single link or the node itself. Even though false positives exist in the connection clusters, the clusters can be classified into different groups with different labels. Compared with the Louvain-based community detection method, the clustering method proposed in the paper shows more superiorability to mine the effective information with confused functional connection matrices (see Figure 8, Figure 9 and Figure 11). In Louvain algorithm, the community is formed according to the largest modularity increment [50]. The modularity is defined as the difference between the connection weights of nodes in the community and the connection weights in a random case. The algorithm cannot make sure that the community of the chosen neighbor is the most appropriate community in which the current node *i* is ought to be. This may lead to instability of the performance. Additionally, it requires the assumption that a random neighbor is likely to be in a good community, which may not be appropriate for the brain networks in Figure 11.

Unlike Louvain, the clustering method proposed in the paper mainly calculates the distance between each connection cluster to classify the nodes into different groups. The classification mode changes with the progress of fatigue or reflects the alerting effect of RADIO. As shown in Figure 8, the active connection clusters labeled as high classes moved from the frontal region to the parieto-occipital region with the accumulation of driver fatigue. Since the brain networks were constructed in alpha band, it reveals a coupling effect between the alpha connection clusters and alpha oscillations themselves. That is to say that the decrease of active level of the connection clusters in frontal region may also correspond to the weakening of motivation and wakefulness and the increase of active level of the connection clusters in the parieto-occipital region can indicate more eye closures and driver drowsiness. Therefore, the clustering algorithm based on functional connectivity can reveal fatigue information. The analysis of alpha oscillations provides a clue for the interpretation of the active cluster movement, in other words, the change of the active level of alpha oscillations in the frontal and parieto-occipital regions may be caused by the active cluster movement. We then took the change of classification mode in Figure 8 as a reference to analyze the alerting effect of RADIO. In DMNR, the most active clusters were distributed in the auditory and frontal areas of the brain. It illustrates that the brain indeed reacts when the drivers are listening to the radio. Acoustic information may enter the brain through the clusters in the auditory area and accompany driving information to be comprehensively processed by the cluster in the frontal region. As the prolonged driving task progressed, only the clusters containing cyan nodes moved to parieto-occipital region in Figure 9, which reflected relatively low-class fatigue information. Particularly, the most active clusters labeled as Classes 3 and 4 remained in the frontal area, after cyan nodes moved to the parieto-occipital region. Because the frontal region is responsible for higher cognitive functions, the motivation and wakefulness of the drivers in the experimental group were probably enhanced during the prolonged driving task. The entropy-based analysis confirmed the movement of connection clusters accompanied with the information content change. As shown in Figure 12, the overall DE values of the experimental group were larger than those of the control group, indicating that the fatigue countermeasure was effective. Entropy measures the average uncertainty of event set to embody information content. Therefore, it revealed the recovery of complexity of brain activity when RADIO was taken. The recovery of activity complexity brought the larger information content. Throughout the control and experimental groups, the trend of DE curves in the frontal and parieto-occipital regions was consistent with the movement of connection clusters. The DE rise of frontal region and decline of parieto-occipital region in the experimental group may be a result of the movement of connection clusters.

Comparing Figure 9 and Figure 12b with Figure 8 and Figure 12a, we draw conclusions about the alerting effect of RADIO. Although the alpha connection clusters and alpha oscillations are coupled with each other to some extent, RADIO relieves fatigue by preventing the active cluster movement from the frontal region to the parieto-occipital region, not the alpha propagation. Making the active clusters stay in the frontal area may just be maintaining alertness.

Note that all the subjects in the experiments were males. In the control group, it requires the subjects to drive a long time without any fatigue countermeasure. We did not recruit female subjects. Even if this study focuses on the methods to investigate the effect of RADIO to maintain driver alertness, the gender factor is not considered. This is a result limitation.

Future work will concern the improvement of localization precision of active clusters. High-density electrode setting will be adopted so that the default network of driving and neural mechanism of alerting effect can be explored in depth.

## 5. Conclusions

In this paper, a new framework to improve the performance of connectivity analysis for RADIO effect exploration has been proposed. Instead of analyzing the network system behavior, dividing into subsets of electrodes obtains a more powerful result. The subsets are classified by the clustering algorithm implemented in brain networks automatically. The quantitative analysis based on DE proves the effectiveness of the method and the active cluster movement after classifying generates the information content change. The alerting effect of RADIO has a relationship with suppressing the active cluster movement from the frontal region to the parieto-occipital region, which is helpful for maintaining driver alertness during prolonged driving. The limitation of this study is that only male subjects participated in the experiments. Whether the effect is adapted to females is unknown.

## Figures and Tables

**Table 1:** Results of the Mann–Whitney U test for differences in global connectivity energy.

Segment	Control Group vs. Experimental Group	
	*Z*	*p*
1	−1.13	>0.05
2	1.04	>0.05
3	1.36	>0.05
4	0.57	>0.05
5	1.17	>0.05
6	1.77	>0.05

**Table 2:** Results of the Mann–Whitney U test for differences in alpha energy ratios.

Segment	Frontal Area vs. Parieto-Occipital Area				Control Group vs. Experimental Group			
	Control Group		Experimental Group		Frontal Area		Parieto-Occipital Area	
	*Z*	*p*	*Z*	*p*	*Z*	*P*	*Z*	*p*
1	−4.02	<0.01	−4.20	<0.01	0.07	>0.05	−1.77	>0.05
2	−3.70	<0.01	−3.88	<0.01	−0.34	>0.05	−0.48	>0.05
3	−3.56	<0.01	−4.30	<0.01	−1.03	>0.05	−0.94	>0.05
4	−3.93	<0.01	−4.20	<0.01	−1.45	>0.05	−1.26	>0.05
5	−3.97	<0.01	−4.11	<0.01	−0.99	>0.05	−0.16	>0.05
6	−3.97	<0.01	−4.39	<0.01	−0.76	>0.05	−0.16	>0.05

**Table 3:** DE values in every segment. * denotes *p* < 0.05. ** represents *p* < 0.01.

Group	Brain Area	DE (Mean ± SD)					
		Segment 1	Segment 2	Segment 3	Segment 4	Segment 5	Segment 6
Control	Frontal	1.90 ± 0.46 **	1.92 ± 0.31 **	2.02 ± 0.55 **	1.89 ± 0.51 **	1.91 ± 0.30 **	1.41 ± 0.36 **
	Parieto-occipital	1.55 ± 0.61 **	2.81 ± 0.62 **	3.03 ± 0.82 *	2.95 ± 0.74 **	2.93 ± 0.50 *	2.76 ± 0.65 **
Experiment	Frontal	6.66 ± 2.30 **	6.09 ± 1.68 **	6.12 ± 1.73 **	5.95 ± 1.19 **	6.37 ± 1.60 **	8.57 ± 4.17 **
	Parieto-occipital	9.76 ± 2.98 **	3.98 ± 1.02 **	4.15 ± 1.28 *	4.25 ± 1.37 **	4.08 ± 1.44 *	4.21 ± 1.15 **

**Table 4:** Results of the Mann–Whitney U test for differences in DE.

Segment	Control Group vs. Experimental Group			
	Frontal Area		Parieto-Occipital Area	
	*Z*	*p*	*Z*	*p*
1	−4.48	<0.01	−4.48	<0.01
2	−4.48	<0.01	−2.96	<0.01
3	−4.48	<0.01	−2.55	<0.05
4	−4.48	<0.01	−2.60	<0.01
5	−4.48	<0.01	−2.37	<0.05
6	−4.30	<0.01	−3.52	<0.01
